# The Impact of COVID-19 on African American Dental Students’ Self-Confidence in Implementing Specific Clinical Treatments

**DOI:** 10.3390/dj14100677

**Published:** 2026-10-18

**Authors:** Brian Laurence, Cherae Farmer-Dixon, Ernest Alema-Mensah, Annitrece Downs, David Miller, Tiffany Wilson, Reginald Salter, Robyn McDaniel, Rueben Warren

**Affiliations:** 1 College of Dentistry, Howard University, 600 W Street, Washington, DC 20006, USA; annitrece.downs@howard.edu (A.D.); reginald.salter@howard.edu (R.S.); 2 School of Dentistry, Meharry Medical College, 1005 Dr. D.B. Todd Jr Blvd, Nashville, TN 37208, USA; cdixon@mmc.edu (C.F.-D.); david.miller@mmc.edu (D.M.); t_diamond_2001@yahoo.com (T.W.); ruebenwarren16@gmail.com (R.W.); 3 Department of Community Health and Preventive Medicine, Morehouse School of Medicine, 720 Westview Drive SW, Atlanta, GA 30310, USA; eamensah@msm.edu

**Keywords:** public health dentistry, COVID-19 pandemic, dental education, online learning, dental education, oral health, HBCU

## Abstract

**Background/Objectives:** Few studies have examined the effects of the COVID-19 pandemic on the clinical readiness of dental students in the provision of care. This study examined whether dental students who graduated during the pre-pandemic period (2019 and before) would have higher self-reported clinical skills confidence than those who graduated during the post-pandemic period (2025 and after) at the only two Historically Black Colleges and Universities (HBCUs) in the United States that teach dentistry. The study hypothesized that the change in educational methods that resulted from the pandemic would result in a reduction in self-reported confidence during the senior year. **Methods:** A cross-sectional online survey was sent to dental graduates from two Historically Black Colleges and Universities in the United States. The questionnaire included a validated 40-item instrument to measure self-reported confidence during senior year in clinical procedures and questions about relevant demographic variables. **Results:** In fixed prosthodontics, confidence decreased significantly for tooth preparation, impressions, try-in of ceramic-fused-to-metal restorations, and cementation of fixed prostheses (*p* < 0.05). Confidence also declined for diagnostic and periodontal procedures, including mucogingival anomaly diagnosis, surgical elimination of periodontal pockets, and management of traumatic dental contacts (*p* < 0.05). The largest decline was seen in the diagnosis and conservative treatment of oroantral communications and fistulas. Of particular clinical and statistical importance, there were significant reductions in self-confidence in the ability to perform tooth extractions and minor oral surgery in medically compromised patients, tooth extractions with separation of roots, and diagnosis and therapy of acute and chronic dental infections (*p* < 0.05). **Conclusions:** The findings provide further evidence of the adverse effect of the COVID-19 pandemic on the African American dental workforce. Given the limited access to care that patients traditionally treated by HBCU dentists have, this study provides evidence that the underserved community may be more adversely affected by the pandemic than previously described.

## 1. Introduction

The COVID-19 pandemic impacted all segments of the global population, including those living in the United States [1,2]. The disproportionate burden of the disease has affected racial and ethnic groups, low-income and elderly populations, and those who suffer from other adverse health conditions [3]. Healthcare workers including health professionals and health auxiliaries were classified as “essential workers,” which meant that they were at greater risk of exposure than others in the general population [4]. Given the proximity of dentists, dental hygienists and dental assistants to their patients, this group of healthcare workers were at substantial risk as they provided oral healthcare to the general population, particularly during the preliminary stages of the pandemic when risk levels for the disease were unclear. The immediate response to the COVID-19 pandemic included the closing of academic institutions, including dental schools, and a transition to virtual learning, disrupting the clinical aspect of training. Many of the formerly hands-on workshops and seminars shifted to online instruction [5,6]. Some of these measures to reduce the risk of disease transmission resulted in a fundamental shift in how dental schools provided didactic and clinical instruction. In response, dental schools made the shift to an increased focus on virtual learning, tele-dentistry, and implementing a variety of changes to the clinical and didactic curricula. It is unclear how these changes have affected future dental education to ensure that dental students graduate with an acceptable level of clinical competence [5,6,7,8,9,10]. Dental schools have returned to in-person learning, but some of the shifts towards virtual learning remain in place, and uncertainty also remains about what post-pandemic curriculum changes are needed to ensure dental students receive the right balance of in-person and virtual learning that ensures the highest standards of clinical readiness.

Few studies have examined the effects of the COVID-19 pandemic on the clinical readiness of dental students in the provision of care [9,11,12,13]. The limited studies published to date on the impact of COVID-19 on dental students’ clinical readiness show a significant change, yet the investigators could not identify any studies that examined the impact in the United States, and in the long run, the effect, if any, this shift in dental education may have had on the overall dental patient population, and particularly on underserved and marginalized communities in the United States who have disproportionally borne the heaviest burden of the COVID-19 pandemic [1,14,15,16]. Studies have already documented the adverse impact of the COVID-19 pandemic on the nursing and medical student pipeline for underserved communities in the United States [17,18,19,20,21]. Given the lack of specific research on dental outcomes and its clinical importance to underserved and marginalized communities, the purpose of this study was to understand the impact of the COVID-19 pandemic on the self-reported perception of clinical skills confidence at the time of graduation of dental students graduating during the pre-pandemic compared to the post-pandemic period at two Historically Black Colleges and Universities (HBCUs) in the United States, Howard University College of Dentistry in the Northeast and Meharry Medical College School of Dentistry in the South. Given the particular importance of African American dentists in treating the underserved African American community in the United States, the goal of this study is to identify any areas of self-reported decreased clinical confidence among HBCU dental students in order to identify additional consequences of the pandemic on this community.

## 2. Materials and Methods

### 2.1. Study Design

Ethical approval was obtained from the Institutional Review Boards of the two participating HBCU dental schools before the data collection process began. We contacted graduates and fourth-year dental students of both participating HBCU dental schools. The inclusion criteria were that participants were eligible for the study if they were graduates of either participating HBCU dental school and provided informed consent.

The investigators categorized 2019 and earlier graduates as the pre-pandemic period respondents, 2020–2024 graduates as pandemic era respondents, and the 2025 fourth-year graduating class as the post-pandemic respondents based on when the lockdown procedures were initiated, remote learning was implemented, when remote learning ended, and when in-person instruction resumed at both participating dental schools. In response to changes required because of the pandemic and the resulting social-distancing requirements, faculty developed multiple instructional videos demonstrating various clinical requirements such as single-tooth preparations and bridge preparations to provide students with visual demonstrations of clinical techniques that could not be practiced in person due to social-distancing requirements. The other HBCU implemented a similar process. These changes, in addition to the general changes that occurred at dental schools across the globe in response to the pandemic by moving from in-person to virtual learning, could adversely affect graduating students’ self-reported confidence in performing specific dental procedures.

Pandemic and pre-pandemic era respondents’ email addresses were located using publicly available information, and they were contacted via email and asked to complete an online questionnaire about their self-confidence in performing dental procedures during the senior year of dental school. Post-pandemic respondents were fourth-year dental students at both HBCUs that were within one to two months of graduation at the time of data collection and were contacted using their corresponding dental school email addresses. The investigators used the 40-item instrument described and used by Ilic et al. in their paper describing the effect of the COVID-19 pandemic on final-year dental students’ self-confidence in performing clinical procedures [11]. The instrument includes five questions from restorative dentistry, five questions from endodontics, five questions from removable prosthodontics, five questions from fixed prosthodontics, ten questions from periodontology, and ten questions from oral surgery. The reliability of the instrument was satisfactory as measured by Ilic et al. with a Cronbach’s α of 0.951. The questionnaire asks respondents to rate their level of self-confidence in performing different dental procedures during their senior year of dental school. Each question was scored using a Likert scale with responses as follows: 1 (no confidence), 2 (little confidence), 3 (moderate confidence), 4 (confident), and 5 (very confident). Demographic information from the participants was also obtained, including age, gender, race, ethnicity, year of graduation from dental school, and dental specialty if available. The questionnaire was available online from 1 November 2024 to 28 August 2025, via a link that included the IRB-approved informed consent document, which explained that the questionnaire contained no unique identifiers that would allow respondents to be identified and that participation was completely voluntary.

### 2.2. Data Analysis

The mean self-confidence score of restorative dentistry, endodontics, removable prosthodontics, fixed prosthodontics, periodontology, and oral surgery for each participant was calculated. Descriptive statistics of the reported self-confidence levels are presented by mean values, standard deviation, and median values. Proportions (in %) were used for the sample structure and response rate. The mean self-confidence scores in each dental field were compared among pre-pandemic, pandemic, and post-pandemic using a one-way ANOVA test with a Tukey post hoc test for pairwise comparisons. The comparison was done for the overall samples as well as for each HBCU individually. The sub-analysis for males and females was done using the same methods. The self-confidence score for each dental procedure question was compared between pre-pandemic and post-pandemic using two independent samples *t*-tests. *p* < 0.05 was defined as statistically significant. SAS version 9.4 was used for data management and analysis.

## 3. Results

Across participating institutions, a total of 288 participants were included in the study, comprising 54 respondents from the pre-pandemic period, 130 from the pandemic period, and 104 from the post-pandemic period (Table 1). Response rates for the groups varied significantly depending on the period and decreased significantly across the post-pandemic (80.0%, *n* = 104), pandemic (20.4%, *n* = 130), and pre-pandemic periods (8.1%, *n* = 54). The distribution by school did not differ significantly across the three time periods (*p* = 0.300).

Many respondents identified as Black/African American in all three periods (77.8% pre-pandemic, 87.6% during the pandemic, and 81.7% post-pandemic). No significant differences were observed in racial composition across three time periods (*p* = 0.198). Sex distribution, however, changed significantly over time (*p* = 0.006 for male vs. female). Female representation increased from 50.0% pre-pandemic to 57.7% during the pandemic and 73.1% post-pandemic, while male representation declined from 50.0% to 26.0% in the post-pandemic period (Table 1).

Participants’ overall restorative self-confidence ratings did not differ significantly across the three time points (*p* = 0.389), with median ratings remaining stable at approximately 4.2–4.4 (Table 2). However, overall self-confidence in endodontics increased significantly in the post-pandemic period compared with both the pre-pandemic and pandemic periods (*p* = 0.004). Similarly, overall self-confidence in fixed prosthodontics showed a significant difference across periods (*p* = 0.048), with a trend toward lower confidence during the pandemic. No significant changes were observed for overall self-confidence in removable prosthodontics, periodontology, or oral surgery.

When stratified by institution, Meharry students demonstrated significant changes in self-confidence in endodontics (*p* = 0.002) and oral surgery (*p* = 0.026), with post-pandemic confidence scores higher than those during the pandemic (Table 3). Confidence in fixed prosthodontics showed a decline during the pandemic (*p* = 0.092). At Howard University, significant differences were observed in self-confidence in removable prosthodontics (*p* = 0.004), with post-pandemic confidence higher than pre-pandemic levels (*p* = 0.005). Self-confidence in other specialty areas at Howard displayed no observable variation across time periods (Table 3).

Among male students, no statistically significant differences in self-confidence were observed across time for any dental specialty, with confidence levels ranging between 3.6 and 4.4 across all three periods (tables stratified by gender are available upon request). In contrast, female students demonstrated a significant change in self-confidence for endodontics across the three time periods (overall *p* = 0.007). Across both sexes, confidence in restorative dentistry remained consistently high, while periodontology and oral surgery tended to have slightly lower but stable confidence ratings.

In accordance with the study hypothesis, the investigators compared participants’ self-confidence levels for all 40 clinical procedures before and after the COVID-19 pandemic and observed a significant decline in self-confidence in 11 clinical procedures in the post-pandemic period (Table 4). We also included the mean values during the pandemic period and provide *p* values for comparing all three groups. In fixed prosthodontics, confidence decreased significantly for tooth preparation, impressions, try-in of ceramic-fused-to-metal restorations, and cementation of fixed prostheses. Confidence also declined for diagnostic and periodontal procedures, including mucogingival anomaly diagnosis, surgical elimination of periodontal pockets, and management of traumatic dental contacts. The largest decline was seen in the diagnosis and conservative treatment of oroantral communications and fistulas (*p* = 0.003). Of particular clinical and statistical importance, there were significant reductions in self-confidence in the ability to perform tooth extractions and minor oral surgery in medically compromised patients, tooth extractions with separation of roots, and diagnosis and therapy of acute and chronic dental infections.

## 4. Discussion

In the study population of mostly African American dentists and dental students from two HBCU dental schools, one from the South and one from the Northeast, the COVID-19 pandemic was associated with a significant reduction in self-reported skills across a variety of clinical areas at the time of graduation. The self-confidence level was significantly reduced for recent graduates for specific procedures in oral surgery, periodontology, and fixed prosthodontics. More specifically, the changes in curriculum and graduation requirements that were implemented due to pandemic restrictions were associated with a reduction in self-reported clinical skills during senior year rather than due to the pandemic itself. This study adds to the sparse literature that examines the effects of the COVID-19 pandemic on the self-reported clinical skill set of dental students and residents. Our findings are consistent with earlier studies that showed an adverse impact of the COVID-19 pandemic and the resulting changes in curriculum and graduation requirements on graduating dental students’ clinical readiness [9,11,12,13]. This study is important because it focuses on African American dental students and African American dental graduates, who have historically and currently disproportionately served African American and low-income populations and who also were disproportionately affected by COVID-19 [22]. The practical relevance of the study is that it highlights another healthcare challenge for African American dentists who already disproportionately serve African American and other underserved populations [22,23]. Specifically, the results of this study are like the results of the study by Ilic et al., in that we used the same standardized instrument used in their study, but that study was focused on a white dental student outside the United States [11]. Like the Ilic study, this study found that recent graduates and graduating dental students lack confidence and do not feel safe in performing specific clinical procedures and will have additional educational needs to address these perceived shortcomings. Both the Ilic et al. study and the present study observed statistically significant reductions in self-reported ability to diagnose mucogingival anomalies because of the COVID-19 pandemic and the resulting educational changes. Hill et al. examined the perceived impacts of the COVID-19 pandemic on health, well-being, clinical competency, and the employment opportunities of dental students in New Zealand [11]. Using a sample size of 94 final-year dental students and open-ended surveys distributed during the dental students’ final semester and then six months after graduation, the authors concluded that post-graduation, the participants felt less confident in clinical competency in six of nine self-reported categories of clinical competency, specifically the areas of fixed prosthodontics, oral medicine, and endodontics. In addition, prosthodontics, oral surgery, and the management of complex cases were areas where participants thought they had less experience because of the COVID-19 pandemic. Seventy-five (75) percent of participants thought the loss of clinical time would harm their clinical competency. Hattar et al. investigated the impact of quarantine during the COVID-19 pandemic on the self-perceived preparedness of dental graduates in relation to cognition, communication, and professional skills using a questionnaire administered to fifth (*n* = 193) and fourth year (*n* = 239) dental students in Jordan [13]. The questionnaire on self-perceived preparedness for dental practice included 17 questions from a previously validated scale that explored a broad range of skills and attributes, and a second category that focused on students’ perceived readiness for dental practice concerning confidence in skills acquired before graduation, preference to be monitored after graduation, readiness for independent practice, and predilection for a well-structured year of residency. The study found that 47.3% of students found it difficult to evaluate new dental materials using an evidence-based approach and lacked sufficient evidence-based knowledge of scientific principles to support their dental practice. In addition, 35.1% of fifth-year dental students were not confident in the skills acquired before graduation, and 64.1% wanted to be mentored or indirectly supervised following graduation. Pandarathodiyl et al. examined the self-perceived preparedness of dental undergraduate students and recent dental graduates for practice during the COVID-19 pandemic in Malaysia [12]. The authors used a cross-sectional web-based questionnaire, the Dental Preparedness Assessment Scale (DU-PAS), administered to new dental graduates (*n* = 210) and final year undergraduate students (*n* = 243) [24,25]. The authors concluded that the final year cohort was significantly less prepared for dental practice compared to the newly graduated cohort. Endodontic treatment on multirooted teeth had the lowest competency (29.8%), then assessing treatment needs of patients needing orthodontics (37.1%). Ilic et al. assessed the level of 2020 final year dental students’ self-confidence (*n* = 115) in performing different dental procedures and compared those results to the self-confidence levels of 2019 final year students (*n* = 100) in Serbia to determine the effect of COVID-19 on dental students’ clinical self-confidence [11]. The authors concluded that the COVID-19 pandemic had a significant impact on final year dental students’ self-confidence, with the students affected by COVID-19 being less confident in eight clinical skills.

D’Emilio et al. examined the impact of COVID-19 on the clinical competency of Advanced Education in General Dentistry (AEGD) residents using self-assessments during the pandemic period and concluded that the COVID-19 pandemic had interrupted clinical learning during the pandemic period [26]. The total sample size of the two cohorts examined over four periods was 771. Over the 2019 to 2021 period, the pandemic affected the residents’ ability to manage advanced periodontal disease, manage patients requiring complicated fixed restorations including implants, perform uncomplicated anterior and posterior endodontic therapy, and communicate case designs with laboratory technicians, among other procedures. The results from the present study reinforce these findings regarding periodontal treatment and fixed prosthodontics.

A study of whether dental and dental hygiene students’ career plans were affected by the COVID-19 pandemic by Garcia et al. concluded that minority students were more likely than white students to report a change in their career plans due to the COVID-19 pandemic [27]. The authors concluded that a potential decrease in the workforce of underrepresented dental providers could have long-term consequences not just for dental education and training programs, but also for the delivery of dental services to underserved communities. The investigators in the present study concur with that statement and build on those concerns by adding the additional perceived shortcomings of clinicians who graduated during the COVID-19 pandemic. The challenges are heightened because the results from our study suggest that these recent dental graduates from the two HBCU dental schools, who have historically and currently practiced in underrepresented areas, may be reluctant to practice in underserved areas because of a lack of confidence in practicing independently.

African Americans represent approximately 13.4% of the United States population, but African American dentists represent less than 4.0% of dentists practicing in the United States [23]. There is a geographical maldistribution of dental professionals across the United States, and these shortages are especially prevalent in rural and low-income communities with higher prevalences of African American residents [23]. In addition, African American dentists are more likely to practice in minority and underserved communities than White dentists and treat a disproportionately high percentage of African American patients [22]. If there are increasing numbers of recent African American dental graduates who perceive that they are lacking the clinical skill sets needed to treat an already underserved population, dental care in these geographical areas may decrease. If dental care is less available and accessible in these areas, untreated dental diseases are likely to increase. In addition, African American dentists report higher educational debt than White dentists, which may affect their decisions about where to practice [22]. Finally, almost half of African American dentists did not complete a residency program before entering private practice, which may also affect their willingness and ability to provide specific services, including extractions when needed [22].

The COVID-19 pandemic has disproportionately affected African Americans and African American HBCU students, and exacerbated what was already a lack of pre-pandemic mentoring and institutional opportunities at HBCUs [21,28]. An analysis of United States Census Bureau unemployment insurance data from January 2018 through December 2021 indicated that, compared with pre-pandemic job flows, the share of workers leaving health care after the pandemic was disproportionately female, and the share of workers entering health care who were female or Black was disproportionately smaller [29]. The authors concluded that the COVID-19 pandemic may have long-lasting implications for workers’ willingness to remain in health care jobs. Lastly, a study that examined the Annual Social and Economic Supplement (ASEC) of the Current Population Survey (CPS) in the United States from 2018 through 2022 observed that the COVID-19 pandemic’s impact on the healthcare workforce disproportionately reduced employment for healthcare workers identifying as Black or Indigenous (Semprini). The present study adds to the mounting data showing the disproportionate effect the COVID-19 pandemic has had on communities of color.

## 5. Conclusions

The current findings are best considered in the context of the study’s strengths and weaknesses. This study included a unique population of African American dentists and dental students in the Northeast and Southern United States and thus has relevance to the African American community and the underserved. In addition, the investigators used questions that were previously tested for validity and reliability. Weaknesses included the limited sample size that prevented further sub-analyses, the information on clinical competency that was based on self-report, and the educational curriculum for both participating HBCUs that was not standardized, which could account for some observed differences in clinical readiness. Finally, the questionnaire asks about self-confidence in clinical skills during the senior year and thus could be affected by recall bias given the lengthier timeline for pre-pandemic graduates. The findings provide further evidence of the adverse effect of the COVID-19 pandemic on the African American dental workforce that can adversely affect the quality of care that dentists who graduated during the COVID-19 pandemic era can provide.

## Figures and Tables

**Table 1:** General characteristics of graduate and D4 respondents.

Characteristics	Pre-Pandemic (*n* = 54)	Pandemic (*n* = 130)	Post-Pandemic (*n* = 104)	*p* Value
School (N, %)				0.300
Howard	20 (37.04)	59 (45.38)	52 (50.00)	
Meharry	34 (62.96)	71 (54.62)	52 (50.00)	
Race (N, %)				0.198
Black/African American	42 (77.78)	113 (87.60)	85 (81.73)	
Asian/Pacific Islander	6 (11.1)	8 (6.20)	8 (7.69)	
White	5 (9.26)	2 (1.55)	5 (4.81)	
Other	1 (1.85)	6 (4.65)	6 (5.77)	
Ethnicity (N, %)				0.134
Hispanic or Latino	3 (5.56)	5 (3.94)	11 (10.68)	
Non-Hispanic or Latino	51 (94.44)	122 (96.06)	92 (89.32)	
Gender (N, %)				* 0.006
Female	27 (50.00)	75 (57.69)	76 (73.08)	
Male	27 (50.00)	54 (41.54)	27 (25.96)	
Non-Binary		1 (0.77)	1 (0.96)	
Dental Specialty (N, %)				0.993
General dentist	34 (65.38)	82 (64.57)	N/A	
Orthodontist	5 (9.62)	14 (11.02)	N/A	
Pediatric dentist	9 (17.31)	21 (16.54)	N/A	
Other specialties	6 (7.69)	13 (7.87)	N/A	

* For male vs. female only.

**Table 2:** Values of self-confidence for specific dental fields in the Pre-Pandemic, Pandemic, and Post-Pandemic groups.

Dental Field	Pre-Pandemic (*n* = 54)		Pandemic (*n* = 130)		Post-Pandemic (*n* = 104)		Overall	Pan vs. Pre	Pan vs. Post
	Mean ± SD	Median	Mean ± SD	Median	Mean ± SD	Median	*p* Value	*p* Value	*p* Value
Restorative	4.17 ± 0.87	4.4	4.21 ± 0.66	4.2	4.08 ± 0.65	4.2	0.389	0.946	0.362
Endodontics	3.59 ± 0.88	3.6	3.46 ± 0.80	3.4	3.80 ± 0.68	3.8	* 0.004	0.552	* 0.003
Removable Prosthodontics	3.60 ± 1.08	3.8	3.47 ± 1.00	3.6	3.69 ± 0.72	3.6	0.214	0.681	0.190
Fixed Prosthodontics	3.74 ± 0.93	3.8	3.41 ± 0.97	3.6	3.40 ± 0.79	3.2	* 0.048	0.059	0.998
Periodontology	3.37 ± 0.82	3.4	3.24 ± 0.85	3.2	3.31 ± 0.68	3.1	0.586	0.573	0.827
Oral Surgery	3.75 ± 0.98	4.0	3.51 ± 0.91	3.5	3.52 ± 0.67	3.5	0.177	0.188	0.999

* *p* values less than 0.05.

**Table 3:** Values of self-confidence for dental fields in the Pre-Pandemic, Pandemic, and Post-Pandemic groups.

	Dental Field	Pre-Pandemic (*n* = 34)		Pandemic (*n* = 71)		Post-Pandemic (*n* = 52)		Overall	Pan vs. Pre	Pan vs. Post	Pre vs. Post
		Mean ± SD	Median	Mean ± SD	Median	Mean ± SD	Median	*p* Value			
**Meharry**	Restorative	4.32 ± 0.85	4.5	3.91 ± 0.87	3.9	4.23 ± 0.54	4.2	0.273	0.784	0.242	0.759
	Endodontics	3.68 ± 0.88	3.5	3.45 ± 0.87	3.7	3.95 ± 0.64	4.0	* 0.002	0.336	* 0.002	0.247
	Removable Prosthodontics	3.96 ± 1.08	4.0	3.01 ± 0.81	3.1	3.86 ± 0.69	4.0	0.873	0.898	0.995	0.873
	Fixed Prosthodontics	4.00 ± 0.79	4.0	3.32 ± 0.99	3.2	3.64 ± 0.66	3.6	0.092	0.158	0.897	0.092
	Periodontology	3.46 ± 0.90	3.5	3.22 ± 0.65	3.0	3.38 ± 0.69	3.3	0.740	0.718	0.942	0.889
	Oral Surgery	4.09 ± 0.83	4.3	3.16 ± 0.97	3.1	3.63 ± 0.62	3.7	* 0.026	0.071	0.782	0.024
**Howard**	Restorative	3.91 ± 0.87	3.9	3.96 ± 0.73	4.0	3.93 ± 0.71	4.1	0.958	0.959	0.979	0.991
	Endodontics	3.45 ± 0.87	3.7	3.48 ± 0.78	3.4	3.65 ± 0.70	3.6	0.429	0.986	0.478	0.581
	Removable Prosthodontics	3.01 ± 0.81	3.1	2.99 ± 0.97	3.0	3.51 ± 0.72	3.5	* 0.004	0.994	* 0.005	0.072
	Fixed Prosthodontics	3.32 ± 0.99	3.2	3.06 ± 1.01	3.0	3.17 ± 0.83	2.9	0.540	0.529	0.807	0.815
	Periodontology	3.22 ± 0.65	3.0	3.14 ± 0.82	3.1	3.23 ± 0.66	3.1	0.806	0.913	0.807	0.998
	Oral Surgery	3.16 ± 0.97	3.1	3.26 ± 0.91	3.2	3.40 ± 0.71	3.3	0.500	0.896	0.655	0.539

* *p* values less than 0.05.

**Table 4:** Self-confidence levels assigned for pre-, post- and pandemic groups for specific dental procedures.

Dental Procedure	Pre-Pandemic	Pandemic	Post-Pandemic	*p* Values		
	Mean ± SD	Mean ± SD	Mean ± SD	Pre vs. Post	Pre vs. Pan	Pan vs. Post
1. Tooth preparation in fixed prosthetics	3.85 ± 1.13	3.67 ± 1.10	3.45 ± 0.98	0.011	0.162	0.945
2. Impressions in fixed prosthetics	3.96 ± 1.02	3.73 ± 1.10	3.70 ± 0.81	0.041	0.097	0.592
3. Try-in of ceramic fused to metal reconstructions	3.83 ± 1.05	3.41 ± 1.18	3.29 ± 1.06	0.001	0.013	0.79
4. Cementing of fixed prosthetic	4.02 ± 0.93	3.84 ± 1.11	3.67 ± 0.97	0.016	0.151	0.89
5. Diagnosis of mucogingival anomalies	3.47 ± 1.10	3.26 ± 1.03	3.19 ± 0.93	0.047	0.112	0.705
6. Surgical elimination of periodontal pockets	2.94 ± 1.06	2.70 ± 1.30	2.55 ± 1.17	0.021	0.118	0.82
7. Diagnosis and elimination of traumatic dental contacts	3.63 ± 1.07	3.50 ± 1.13	3.27 ± 0.94	0.015	0.237	0.048
8. Tooth extractions with separation of roots	3.68 ± 1.38	3.36 ± 1.36	3.29 ± 1.12	0.029	0.078	0.663
9. Diagnosis and therapy of acute and chronic dental infections	3.98 ± 0.88	3.72 ± 0.98	3.62 ± 0.91	0.009	0.047	0.788
10. Tooth extraction and minor oral surgery in medically compromised patients	3.74 ± 1.18	3.39 ± 1.21	3.42 ± 1.04	0.041	0.039	0.42
11. Diagnosis and conservative therapy of oroantral communication and fistulas	3.28 ± 1.09	2.71 ± 1.23	2.75 ± 1.09	0.003	0.002	0.399

## Data Availability

The raw data are available upon request from the corresponding author.
